# In Vitro Recombination of Non-Homologous Genes Can Result in Gene Fusions that Confer a Switching Phenotype to Cells

**DOI:** 10.1371/journal.pone.0027302

**Published:** 2011-11-11

**Authors:** Richard A. Heins, Jay H. Choi, Takayuki Sohka, Marc Ostermeier

**Affiliations:** Department of Chemical & Biomolecular Engineering, Johns Hopkins University, Baltimore, Maryland, United States of America; Texas A&M University, United States of America

## Abstract

Regulation of protein activity is central to the complexity of life. The ability to regulate protein activity through exogenously added molecules has biotechnological/biomedical applications and offers tools for basic science. Such regulation can be achieved by establishing a means to modulate the specific activity of the protein (i.e. allostery). An alternative strategy for intracellular regulation of protein activity is to control the amount of protein through effects on its production, accumulation, and degradation. We have previously demonstrated that the non-homologous recombination of the genes encoding maltose binding protein (MBP) and TEM1 β-lactamase (BLA) can result in fusion proteins in which β-lactamase enzyme activity is allosterically regulated by maltose. Here, through use of a two-tiered genetic selection scheme, we demonstrate that such recombination can result in genes that confer maltose-dependent resistance to β-lactam even though they do not encode allosteric enzymes. These ‘phenotypic switch’ genes encode fusion proteins whose accumulation is a result of a specific interaction with maltose. Phenotypic switches represent an important class of proteins for basic science and biotechnological applications *in vivo*.

## Introduction

The regulation of cellular protein activity occurs through many mechanisms including the regulation of production (i.e. transcription/translation), the targeting to specific cellular compartments, the interaction with other molecules (i.e. inhibitors, activators, and allosteric effectors) and the regulation of degradation. The direct regulation of protein activity at the protein level through allosteric effects offers two important advantages: regulation does not depend on any other cellular components besides the effector molecule and the regulation can be executed on much shorter time scales – essentially instantaneously. These advantages have motivated the engineering of synthetic switchable proteins for biotechnological, biomedical and basic science applications [1].

One approach to the engineering of such protein switches is to create fusions of proteins having the prerequisite input and output functions of the desired switch. The key design question for this approach is how to fuse two proteins such that binding of the effector to the input domain modulates the activity of the output domain. We have pursued a directed evolution approach to this design problem, creating libraries of random insertions of one domain into the other and using random circular permutation of the inserted domain to vary the fusion location [2], [3], [4]. Such an approach requires a well-designed selection/screening method in order to find those rare protein switches among the majority of constructs that lack effector-dependent activity. Genetic selections are a powerful tool in this regard. For the selection of switches, one needs to connect the modulation of the switches activity to a selectable phenotype. However, since regulation of cellular protein activity can occur by many mechanisms other than effector-induced modulation of a protein's specific activity, such selections might result in the identification of gene fusions that confer switching behavior to cells by means other than allostery.

We have previously described the use of a genetic circuit that functions like a band-pass filter for enzyme activity to isolate allosteric β-lactamase enzymes [5]. We subjected a library in which random circular permutations of TEM1 β-lactamase gene were inserted in place of codon 317 of the gene encoding *E. coli* MBP to a two-tier selection designed to identify allosteric β-lactamases that had maltose as an effector. In the first tier, the library was challenged to grow on plates containing high concentrations of ampicillin (Amp) and 10 mM maltose. Cells surviving this positive selection were recovered *en masse* and, in the second tier of the selection, challenged to grow in the absence of maltose under band-pass genetic selection conditions. This unusual type of selection, which was created using a synthetic gene network, allows the researcher to select for cells that have β-lactamase activity between two threshold values, much like an electronic band-pass filter is designed to function to isolate frequencies in a desired range. Selection against cells with high β-lactamase activity results from placing a tetracycline resistance gene downstream from the *ampC* promoter, which is induced by low levels of β-lactam antibiotics, and challenging the bacteria to grow in the presence of tetracycline. In this way, cells with high β-lactamase activity degrade all the β-lactam antibiotic, lack tetracycline resistance and can be selected against. This second tier selection was performed in the absence of maltose to select for those library members that confer low levels of β-lactamase activity in the absence of maltose. Of the 34 colonies that survived the second selection, four contained allosteric β-lactamases that had maltose as an effector. This allostery was established by *in vitro* experiments on the purified protein [2]. The remaining 30 colonies did not appear to contain allosteric enzymes as judged by colorimetric assays on cell lysates for β-lactamase activity in the presence and absence of maltose. However, most of these colonies were not false positives for conferring a switching phenotype to cells, as the gene fusions of these library members conferred to *E. coli* an increase in Amp resistance in the presence of maltose – the phenotype that should be necessary to survive both tiers of the selection. We show here that all of these proteins, which we describe as phenotypic switches, accumulate at higher concentrations in the cell in the presence of maltose. We characterize this phenomenon by examining the cellular accumulation, enzymatic activity, and melting temperature of these phenotypic switches. Evidence indicates that this phenotype manifests not from effects on transcription but rather from the specific interaction of maltose with the fusion protein that results in increased accumulation of the protein.

## Results

### Identification of phenotypic switches and a control non-switch

Using a two-tiered genetic selection designed to identify allosteric β-lactamases that are activated by maltose, we isolated 34 library members from a library in which random circular permutations of TEM1 β-lactamase gene were inserted in place of codon 317 of the gene encoding *E. coli* MBP [5]. Four library members harbored enzymes with the desired allosteric property. The lysates of the remaining 30 colonies exhibited β-lactamase activity that did not change upon the addition of maltose. However, 28 of these library members had a >2-fold higher ampicillin minimum inhibitory concentration (MIC_Amp_) when the cells were grown in liquid culture in the presence of maltose compared to its absence. Maltose did not alter the MIC_Amp_ of control cells expressing wildtype BLA, less-active mutants of BLA or no β-lactamase gene. From initial attempts to sequence these library members, it was determined that all 28 contained more than one library plasmid (a not uncommon occurrence in large libraries [6]). Therefore, plasmid DNA from each clone was isolated and transformed into DH5α-E cells. Selection at low Amp concentrations facilitated the isolation of the plasmids that conferred the desired maltose-dependent MIC_Amp_ phenotype, as most of the “extra” plasmids were comprised of out-of-frame insertions of the *bla* gene. Subsequent sequencing revealed 13 unique sequences (Table 1) with many sequences isolated multiple times.

**Table 1:** Sequence of fusion proteins.

Protein	Protein Sequence^a^
MBP317-347	MBP[1-316]-BLA[170-286]-DKS-BLA[24-170]-MBP[318-370]
Ph1	MBP[1-316]-BLA[263-286]-DKS-BLA[24-263]-MBP[318-370]
Ph3	MBP[1-316]-BLA[112-286]-DKS-BLA[24-114]-T-MBP[319-370]
Ph5	MBP[1-316]-BLA[267-286]-DKS-BLA[24-266]-MBP[318-370]
Ph7	MBP[1-316]-BLA[24-286]-MBP[318-370]
Ph8	MBP[1-316]-BLA[264-286]-DKS-BLA[24-263]-MBP[318-370]
Ph12	MBP[1-316]-BLA[260-286]-DKS-BLA[24-263]-MBP[318-370]
Ph14	MBP[1-316]-BLA[286]-DKS-BLA[24-286]-MBP[318-370]
Ph16	MBP[1-316]-BLA[24-286]-DKS-BLA[24]-MBP[318-370]
Ph17	MBP[1-316]-BLA[181-286]-DKS-BLA[24-191]-MBP[318-370]
Ph19	MBP[1-316]-BLA[194-286]-DKS-BLA[24-194]-MBP[318-370]
Ph24	MBP[1-316]-BLA[168-286]-DKS-BLA[24-168]-E-MBP[319-370]
Ph27	MBP[1-316]-BLA[113-286]-DKS-BLA[24-113]-MBP[318-370]
Ph28	MBP[1-316]-BLA[40-286]-DKS-BLA[24-39]-MBP[318-370]
c4	MBP[1-316]-BLA[24-286]-DK-T-MBP[319-370]

a Based on gene sequencing.

These 13 library members were retransformed into RH22 cells and their ability to confer maltose-dependent MIC_Amp_ tested in 5 ml liquid culture. All conferred a maltose-dependent resistance to Amp with maltose increasing the MIC_Amp_ by 4-fold to 64-fold (Table 2). We refer to these library members as phenotypic switches, since they confer a MIC_Amp_ switching phenotype that depends on maltose.

**Table 2:** The effect of maltose on MIC_Amp_ and protein expression.

	MIC_Amp_ (µg/ml)^b^			Accumulation^c^	
Protein^a^	− Maltose	+ Maltose	Ratio^d^	Ratio^d^	Stdev^e^
None^f^	1	1	1	2.4	0.2
MBP	1	1	1	1.0	0.1
BLA	8192	8192	1	1.3	0.1
MBP317-347	16–32	512	16–32	31.3	4.5
Ph1	32	512–1024	32–64	14.6	3.1
Ph3	256	1024–2048	4–8	10.7	1.0
Ph5	16–32	256–512	8–32	17.6	5.9
Ph7	32–64	1024	16–32	25.7	6.5
Ph8	32	512–1024	16–32	19.2	7.5
Ph12	64	1024–2048	16–32	38.9	10.1
Ph14	128	2048	16	12.4	6.8
Ph16	1024	4096	4	5.4	2.1
Ph17	32	1024	32	49.2	7.9
Ph19	256	2048	8	14.7	2.2
Ph24	4–8	128	32	21.5	4.7
Ph27	256	2048	8	11.1	3.7
Ph28	64	1024–2048	16–32	22.7	7.2
c4	1024–2048	2048	1–2	5.3	5.0

a Protein expressed from plasmid pDIMC8 in RH22 cells.

b MIC_Amp_ was determined in triplicate. For proteins with a MIC_Amp_ range, this indicates the range from the three experiments (e.g. Ph5 was found to have a MIC_Amp_ in the absence of maltose of 16 µg/ml in some trials and 32 µg/ml in other trials).

c Accumulation ratio determined from quantitative image analysis of western blots probed using polyclonal anti-BLA antibodies (except for MBP which was probed with anti-MBP antibodies).

d (With maltose)/(without maltose).

e Standard deviation calculated from three independent experiments.

f No pDIMC8 plasmid was present. The protein monitored by western blot was chromosomally encoded MBP.

Ph8 was chosen as a representative phenotypic switch for further characterization. For comparison, we desired a control fusion protein of BLA and MBP that conferred ampicillin resistance in a maltose-independent fashion. Such library members were readily identified by screening colonies after the first tier of the selection. We sequenced one of these library members and named it c4 (Table 1). As an additional control we chose MBP317-347, one of the allosteric enzymes previously isolated from the same library [2], [5].

### Maltose increases the steady state protein levels of soluble phenotypic switches but not through transcriptional effects

**Figure 1:**
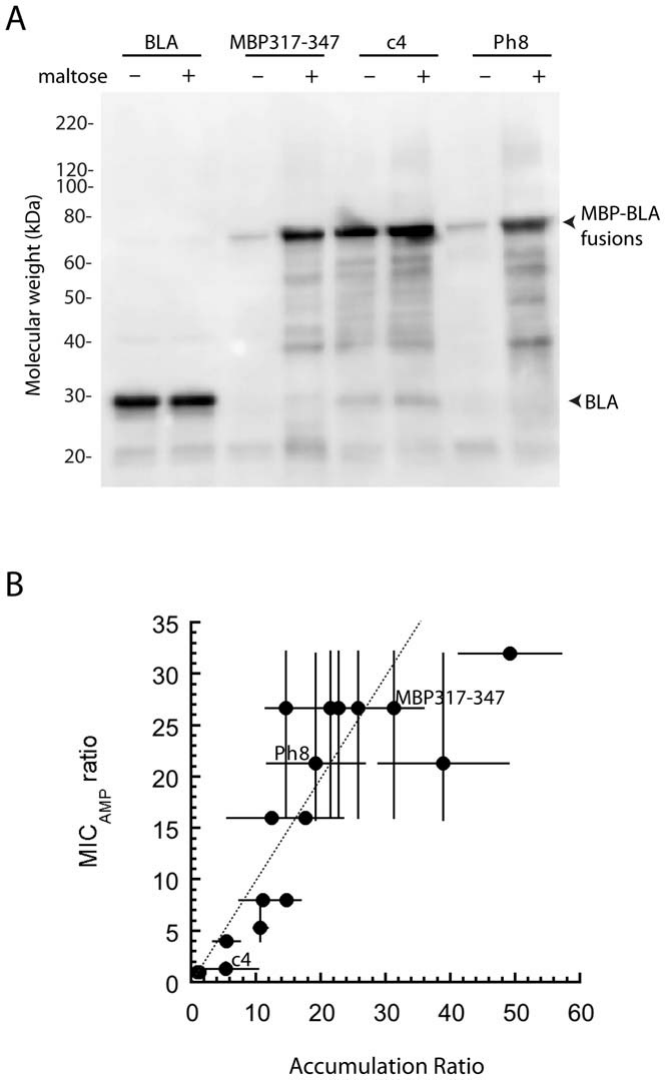
Characterization of phenotypic switches. (A) Western blot using anti-BLA antibodies of the soluble fraction of cells expressing BLA (lower arrow, ∼30 kDa), MBP317-347, c4, and Ph8 (all upper arrow, ∼70 kDa) cultured in the presence (+) and absence (−) of 5 mM maltose. (B) Correlation between the accumulation ratio (+maltose/−maltose), as measured by western blot, and the MIC_Amp_ ratio (+maltose/−maltose) of cell expressing the 13 phenotypic switches, MBP317-347, c4 and BLA. The dotted line is y = x. Error bars represent the standard deviation (N = 3).

Unlike MBP317-347, which exhibits a 150-fold increase in β-lactamase activity when maltose is added to lysates expressing this allosteric switch [2], the β-lactamase activity in the lysates of these 13 members was maltose-independent. This indicated that these proteins were not allosteric enzymes. A possible explanation for how these genes could confer an increase in MIC_Amp_ in the presence of maltose was increased accumulation of active protein. Thus, the effect of maltose on the accumulation of soluble fusion protein was examined by western blot using anti-BLA antibodies. Accumulation levels of fusion protein in the presence and absence of maltose were determined under the same culture conditions as the MIC assays. The accumulation of phenotypic switch Ph8 but not the control BLA was markedly increased by the presence of maltose in the growth media (Fig. 1A). Interestingly, allosteric switch MBP317-347 also showed a dramatic increase in accumulation in the presence of maltose (Fig. 1A), raising the question of how much of its 16- to 32-fold increase in MIC_Amp_ in the presence of maltose is a result of allosteric effects and how much is a result of increased accumulation of the protein in the cell. Maltose increased the accumulation of all 13 selected members (Table 2). A significant correlation between the increase in protein levels and the increase in MIC_Amp_ in the presence of maltose (Fig. 1B) suggested that increased accumulation of the fusion in the presence of maltose is the major cause of the maltose-dependent MIC_Amp_ phenotype. The cause of the increased accumulation is unlikely to be transcriptional in nature, since maltose did not increase the accumulation of MBP or BLA when the respective genes were downstream from the *tac* promoter – the same promoter that controlled the expression of the switches (Table 2). Maltose increased the accumulation of MBP expressed from the chromosome as expected [7], but not when MBP was expressed under the *tac* promoter on the plasmid (Table 2). C4 exhibited a highly-variable but low increase in accumulation in the presence of maltose; however, this is equal to or lower than the increase observed with any of the phenotypic switches.

### The increase in soluble Ph8 and Ph14 does not correlate with a decrease in insoluble protein

To investigate whether the absence of maltose caused the phenotypic switches to misfold and aggregate, we analyzed the soluble and insoluble fractions of Ph8 and Ph14 by western blots. Samples from cultures grown in the absence of maltose showed very low amounts of soluble or insoluble protein, whereas samples from cultures containing maltose showed an increase in both the soluble and insoluble fractions (data not shown). Thus, the increase in protein accumulation in the presence of maltose does not appear to result from prevention of misfolding and aggregation.

### Proteins chosen for *in vitro* characterization

For the remaining studies, Ph8 and c4 were selected for purification and *in vitro* characterization. TEM1-BLA, MBP and MBP317-347 [2] were purified as control proteins. We successfully purified Ph8 and c4 using an amylose affinity column. This result indicates that the insertion of BLA did not abolish their ability to bind maltose since maltose was used to elute these proteins from the column. This maintenance of maltose binding is consistent with other hybrid proteins created by inserting circularly permuted BLA in place of MBP residue 317 [2].

### Neither Ph8 nor c4 are allosteric enzymes

We determined the Michaelis-Menten kinetic parameters for Amp hydrolysis of purified Ph8 and c4 at 37°C (Table 3). The *K*_m_ and *k*_cat_ for Amp for both proteins did not change upon the addition of 5 mM maltose. c4 has similar catalytic properties to TEM1-BLA [8], [9], which is not too surprising considering the BLA domain in this protein is essentially wild-type (i.e. it is not circularly permuted) (Table 1). Ph8 has a 10-fold higher *K*_m_ compared to wild-type BLA, but a wild-type *k*_cat_. These kinetic studies confirm that neither Ph8 nor c4 is an allosteric enzyme with maltose as an effector.

**Table 3:** Kinetic constants for the hydrolysis of ampicillin^a^.

Protein	K_m_ (µM)		*k*_cat_ (S-1)		*k*_cat_/K_m_ ratio^c^
	− Maltose	+ Maltose	− Maltose	+ Maltose	
Ph8	730±120	700±100	1870±220	1950±110	1.1±0.3
c4	73±4	77±8	2280±120	2350±110	1.0±0.1
BLA^b^	32	−	1050	−	−

a Assays were at 37°C in 10 mM phosphate buffer (pH 7.0) with or without 5 mM maltose.

b At 30°C in 50 mM phosphate buffer (pH 7.0) [9]. Previous studies have shown that BLA enzyme activity is unaffected by maltose [4].

c (*k*_cat_/K_m_)_+maltose_/(*k*_cat_/K_m_)_−maltose_.

### Maltose-binding increases the melting temperature of the Ph8 and c4 by equal amounts

The insertion of BLA into MBP can be a destabilizing event that may result in a fusion protein with a lower melting temperature than either of the two parental proteins. Conversely, the binding of maltose to MBP is a stabilizing interaction with an increase in melting temperature by about 8°C [10]. If the melting temperature of the fusion was near or below the growth temperature in the absence of maltose, but increased above the growth temperature in the presence of maltose, this could account for the increased accumulation in the presence of maltose.

Temperature-induced unfolding experiments were conducted on MBP, BLA, MBP317-347, Ph8 and c4 to determine the melting temperature (T_m_) in the presence and absence of maltose. We measured changes in ellipticity as a function of temperature by circular dichroism spectroscopy. The presence of maltose increased the T_m_ of MBP by 7.9°C (Table 4), in good agreement with published values [10]. As expected, maltose had no effect on the T_m_ of BLA, and our value for T_m_ (∼52.5°C) closely matched that of a previous study (50.1°C) [11]. MBP317-347, Ph8 and c4 all had significantly lower T_m_ values compared to MBP (a loss of between 15 to 20°C), indicating that the insertion of the BLA domain negatively affected their melting temperature. The low T_m_ of MBP317-347 and Ph8 in the absence of maltose (only 8–10°C above growth conditions of the bacteria) suggested that low thermal stability could play a role in the reduced accumulation of these proteins. The addition of maltose increased the T_m_ for both of these proteins by about 10°C, offering a potential mechanism for the increased accumulation of these proteins. However, c4's melting temperature in the presence and absence of maltose was the same as the values for MBP317-347 and Ph8. Since Ph8 and c4 had equivalent T_m_'s yet only Ph8 conferred a switching phenotype, a ligand-induced increase in melting temperature does not necessarily confer a switching phenotype and may not be the mechanism by which Ph8 confers a switching phenotype.

**Table 4:** Melting temperatures of proteins.

	T_m_ (°C)^a^	
Protein	− Maltose	+ Maltose
MBP	64.4	72.3
BLA	52.4	52.6
MBP317-347	44.6	54.3
Ph8	47.2	57.5
c4	46.4	56.4

a T_m_, temperature at transition midpoint, determined by temperature-induced unfolding monitored by CD spectroscopy. All temperatures are ±0.2°C.

### Osmolyte-protein interactions are not the cause of increased production of phenotypic switches

Maltose and sucrose are known osmolytes [12] a class of molecules known to shift the conformational equilibrium proteins towards a folded state due to solvation interactions with the peptide backbone. Osmolyte interactions are weak, typically requiring millimolar to molar concentrations to effectively influence protein folding [13]. Since the switching phenotype is observed in experiments with 5 mM maltose in the growth media, it was possible that this interaction was partially or wholly responsible for the increased production of the phenotypic switches. However, the addition of 5 mM maltose did not increase the melting temperature of BLA, indicating that 5 mM maltose does not appreciably stabilized BLA.

To determine whether maltose affected the phenotypic switches by a specific ligand-protein interaction or a more general osmolyte effect, we compared the effect of maltose and sucrose on the production of MBP317-347, Ph8 and two variants of MBP317-347 (5–7 and MBP317-347-W340A). 5–7 is a quadruple mutant of MBP317-347 that has both maltose and sucrose as allosteric effectors [2]. This switch retains micromolar affinity for maltose (apparent K_d, maltose_ = 23 µM), and its sucrose affinity has increased by more than 10^6^-fold relative to MBP317-347 (apparent K_d, sucrose_ = 0.7 µM) [2]. MBP317-347 has fairly strong maltose affinity (*K*_d_ = 0.5 µM [2]), while the introduction of the W340A mutation is known to reduce the maltose affinity of wild-type MBP by about 1000-fold [14]. MBP317-347 was chosen as the example phenotypic switch for these studies instead of Ph8 because (1) MBP317-347 is more characterized and (2) the mutations that confer binding to sucrose were identified in MBP317-347 and it was uncertain whether such mutations would work in Ph8.

**Figure 2:**
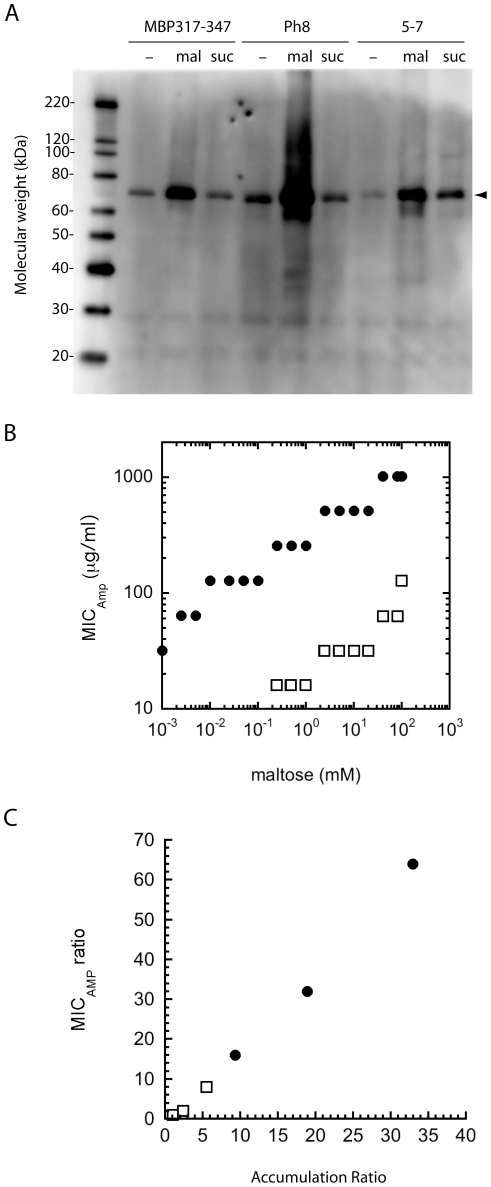
Effect of ligand concentration on MIC_Amp_ and accumulation of phenotypic switches. (A) Western blot using anti-BLA antibodies of the soluble fraction of cells expressing MBP317-347, Ph8 and sucrose switch 5–7 (arrow, ∼70 kDa). Cells were cultured with maltose (mal), sucrose (suc), or neither sugar (−). (B) Correlation between the MIC_Amp_ and the concentration of maltose added to the culture for cell expressing MBP317-347 (•) or MBP317-347(W340A) (□). (C) Accumulation levels dictate the MIC_Amp_. Correlation between the accumulation ratio (+maltose/−maltose), as measured by western blot, and the MIC_Amp_ ratio (+maltose/−maltose) of cell expressing MBP317-347 (•) or MBP317-347(W340A) (□). Different accumulation ratios were obtained by culturing the cells in the presence of different concentrations of maltose.

We cultured cells expressing these proteins in the presence and absence of different concentrations of maltose and sucrose. The accumulation of soluble protein was compared by western blot. The results indicate that a specific ligand-protein interaction and not an osmolyte effect causes the increased accumulation. Only 5–7 – the one switch with high affinity for sucrose – showed an increase in production in the presence of sucrose (Fig. 2A). The addition of the W340A mutation to MBP317-347 dramatically impaired maltose's ability to increase the MIC_Amp_ of cells expressing the switch, requiring about 1000-fold higher maltose concentrations for similar MIC_Amp_ increases (Fig. 2B). In addition, both MBP317-347 and MBP317-347/W340A accumulation increased with maltose concentration in a dose-dependent manner (Fig. 2B). A detectable MIC_Amp_ increase for cells expressing MBP371-347 required only 1 µM maltose (Fig. 2C), which is inconsistent with the high concentrations needed for an osmolyte effect. These experiments strongly suggest that a specific ligand-protein interaction is essential for enhanced accumulation and that any beneficial folding effects conferred by these osmolytes are minimal with respect to the accumulation of protein.

## Discussion

With one exception, the phenotypic switch genes conferred a MIC_Amp_ above 200 µg/ml in the presence of maltose (Table 1). This is consistent with the first tier of the selection that was used to isolate them. However, in the absence of maltose, 5 of 13 of the phenotypic switches conferred an MIC_Amp_≥128 µg/ml – higher than expected since the second tier should select for library members that confer an MIC_Amp_ of 16, 32 or 64 µg/ml [5]. We speculate that the presence of two plasmids in the initially selected library members – one encoding a phenotypic switch and one encoding a non-functional β-lactamase – conferred a larger difference in the maltose-dependent MIC_Amp_ through selective pressure on the two plasmids. In the second tier of the selection, in which cells were required to have low β-lactamase activity to survive, cells in which the copy number of the plasmid encoding the non-functional β-lactamase increased relative to that encoding the phenotypic switch would have a selective advantage for growing under the conditions of this selection. Such a change in the relative copy number of the two library plasmids could occur for stochastic reasons during cell division and lower the amount of cellular β-lactamase activity enough to permit survival. This theory is further supported by the fact that the maltose-dependent differences in MIC_Amp_ were greater for some of the originally isolated library members bearing both plasmids than for cells bearing only the plasmid encoding the phenotypic switches.

The mechanism by which maltose causes an increase in the accumulation of the phenotypic switches is not clear. Maltose had no effect on accumulation of BLA or MBP. This finding is not consistent with any mechanism involving changes in transcription/translation. Furthermore, a transcriptional mechanism would be expected to result in an equal magnitude switching phenotype for all fusions in the library, not a variable switching phenotype for just a select few. The effect of mutations designed to alter sugar specificity and affinity indicate that a specific sugar-protein interaction is required for the increase in production. This evidence discounts a role for the osmolyte effect and also does not support a mechanism involving changes in transcription/translation.

Ligand-induced thermodynamic stabilization is a possible mechanism for the observed increase in cellular accumulation in the presence of ligand. For example, Foit et al. found that when a variety of test proteins were inserted into BLA, the test protein's thermostability predicted the penicillin V resistance conferred by the fusion [15]. Although the study did not address ligand binding per se, mutations in the test protein that increased thermostability also increased the accumulation level of the protein and the conferred MIC relative to that of fusions made with the wild type protein. When the insert protein was immunity protein 7, an increase in accumulation level resulted in an equivalent increase in MIC (R^2^ = 0.60). A mutation causing a 6 kJ/mol increase in stability (ΔΔG°) caused a 2.7-fold increase in accumulation and a 2.7-fold increase in MIC, on average. However, for other proteins, an equivalent stabilization resulted in a considerably smaller increase in MIC. For granulocyte stimulating factor, a 10 kJ/mol increase in stability only resulted in about a 1.4-fold increase in MIC. Thus, for fusions of proteins with BLA, the relative effect of stabilization on accumulation levels and MIC depends on the identity of the fusion partner (and likely the topography of the fusion).

Although we have not yet measured the thermodynamic stability of the phenotypic switches, ligand-induced thermodynamic stabilization by itself cannot explain our results. First, MBP is thermodynamically stabilized −35 kJ/mol by binding to maltose [16], yet, its accumulation was not altered by growing the cells in the presence of maltose. Second, any fusion protein in which the MBP domain retains the ability to bind maltose would be thermodynamically stabilized by maltose-binding, yet most such fusions are not phenotypic switches. Confounding factors include the fact these two domain proteins are unlikely to fit the simple two-state model of protein folding and the high likelihood that the thermodynamic stability of the BLA domain of most fusions may be partially or even entirely independent of the state of MBP domain. Perhaps, phenotypic switches are comprised of the small subset of fusions in which the thermodynamic stability of the BLA domain is linked to the MBP domain – a hypothesis that we intend to test in future experiments.

Any mechanism that increases the rate of achieving the folded state or decreases the rate at which the protein is degraded would cause increased accumulation. Thus, ligand-induced folding and a ligand-induced decrease in the rate of proteolytic degradation of the phenotypic switches are additional, non-mutually exclusive mechanisms that could contribute to a ligand-dependent increase in accumulation. Regarding the latter mechanism, the cpBLA domains found in the phenotypic switches might expose unstructured loops (known protease substrates [17], [18]) due to distortion of the wild-type fold upon fusion to MBP. Maltose-binding to the fully folded protein could modulate the availability of these or other proteolytic-sensitive sites on the protein though maltose-induced conformational changes in the protein. Such a protein could be considered to be a type of allosteric protein with maltose-binding causing conformational changes that affect the distant, proteolytic sensitive site. Alternatively, maltose binding might enhance the folding rate such that the phenotypic switches can fold to a proteolytic resistance form before they acted on by a protease. Maltose might increase the folding rate of the protein through the stabilization of intermediates along the folding pathway. If such intermediates sequester protease-susceptible sites, this could explain the increased accumulation of the protein in the presence of maltose.

Regardless of the mechanism, the prevalence of phenotypic switches in our library of non-homologously recombined genes has important implications for switch construction and switch application. If the application of the switch is designed to be inside the cells (e.g. as a cellular reporter of effector concentration or as a selective therapeutic protein), then the proteins do not necessarily need to behave as allosteric switch *in vitro* to be useful. Thus, these phenotypic switches represent an important class of switch proteins for *in vivo* applications such as protein therapeutics. For example, we recently engineered a switch that renders human colon and breast cancer cells susceptible to a prodrug in a cancer marker-dependent fashion [19]. The switch (called Haps59) consisted of a fusion between a yeast cytosine deaminase (an enzyme that can activate a prodrug) and the CH1 domain of the human p300 protein, a domain that can bind to the cancer marker HIF-1α. The gene encoding this switch selectively conferred increased susceptibility to the prodrug in cells that accumulated HIF-1α. This increased prodrug susceptibility correlated with increased accumulation of Haps59, analogous to how ampicillin resistance correlated with increase switch accumulation in the data reported here. Haps59 is thus a phenotypic switch.

Our isolation of many phenotypic switches in the work presented here suggests that this type of switches is at least as common as allosteric switches in libraries created by our recombination strategy. However, phenotypic switches can only be readily identified through selection strategies that select for the switching phenotype. For example, screens involving assays of cell lysates for effector-dependent activity, like we had previously used to identify maltose-activated β-lactamases, will miss these potentially useful proteins [2], [3], [4]. On the other hand, if switches with *in vitro* switching activity are desired, selections based on conferring a switching phenotype to cells warrant a secondary screen to evaluate switching in an *in vitro* setting in order to eliminate switches that function solely through an effector-dependent increase in switch accumulation.

## Materials and Methods

### Strains

The phenotypic switches were discovered using *E. coli* strain RH22 [5]. Electromax DH5α-E electro-competent cells were purchased from Invitrogen. The *malE* knockout strain PM9F′ [20] was used for protein purification of Ph8 and c4. DH5α-E was used for the production of BLA.

### Selection, isolation and identification of phenotypic switches and control fusion

Selection of cells from a two-tiered selection system was described in Sohka et al. [5]. The MIC_Amp_ of these colonies (34 total) was determined in the presence and absence of maltose. Colonies with >2-fold MIC_Amp_ difference in the liquid culture MIC_Amp_ assays (see below) were sequenced; the sequencing data was poor in every case, suggesting the presence of multiple pDIMC8-derived plasmids. Purified plasmid DNA from a standard miniprep (concentration ∼200–300 ng/µL) for each colony was diluted 1000 fold into water and 1 µL was transformed into DH5α-E cells (Invitrogen). The transformed bacteria were spread on LB-agar plates supplemented with chloramphenicol (Cm) (50 µg/mL), isopropyl-*β*-D-thiogalactopyranoside (IPTG) (300 µM), maltose (5 mM) and Amp (4 µg/mL) and incubated overnight at 37°C. The sequencing of plasmid DNA from the resulting colonies did not have the aforementioned problems.

The control fusion protein c4 was isolated by screening colonies that survived the first tier of the switch selection procedure (i.e. these library members have the ability to grow at high concentrations of Amp in the presence of maltose). The effect of maltose on the MIC_Amp_ was determined using the liquid culture MIC_Amp_ assays (see below). Library members that did not show a difference in the MIC_Amp_ were sequenced. Most members contained a frame-shift mutation terminating the chimeric protein shortly after the cpBLA insertion site; one member, however, had an in-frame insertion. This member was named c4.

### Liquid culture MIC_Amp_ assays

The optical density at 600 nm (OD_600 nm_) was measured for each of the 10 ml overnight culture of cells expressing the phenotypic switches in tryptone broth (10 g/L tryptone and 10 g/L NaCl). Tryptone broth was used instead of LB for these experiments since low-levels of maltodextrans have been detected in yeast extract [2]. In each culture tube, approximately 1×10^6^ colony forming unit (based on OD_600 nm_) from the overnight culture was added to 5 ml of tryptone medium containing Cm (50 µg/ml), IPTG (300 µM), different concentrations of Amp, and either the presence or absence of 5 mM maltose. The tubes were incubated at 37°C in a shaker incubator. After 18 hours of incubation, 200 µl was transferred to clear-bottom 96-well plates and the OD_600 nm_ was determined using a Molecular Devices SpectraMax 386 plus. The MIC for Amp was defined as the lowest concentration of Amp at which the OD_600 nm_ was <3% of the OD_600 nm_ in the absence of Amp.

### Quantification of accumulation by western blotting

From the cultures used to determine MIC_Amp_ (see above), 6.8×10^8^ cells (based on OD_600 nm_) were collected and pelleted by centrifugation at 4500 g for 10 min at 4°C and resuspended in 250 µL of Bugbuster (Novagen) supplemented with rlysozyme (Novagen, 1 KU per 1 mL), benzonase nuclease (Novagen, 25 U per 1 mL) and protease inhibitors (Sigma). Cells were lysed for 30 min at room temperature, centrifuged at 20,000 g for 30 min at 4°C, then the soluble fraction was transferred into a new 1.5 ml tube. The soluble fractions were loaded onto a 4–12% Bis-Tris Gel (Invitrogen) and electrophoresed for 45 minutes at 190 V. Proteins were transferred to PVDF membrane (Bio-Rad) at 15 V for 30 minutes using a semi-dry transfer cell (Bio-Rad).

The membrane was processed following the guidelines for the SNAP-ID system (Millipore). The membrane was blocked with 30 mL 0.2% milk in Tris buffered saline, 0.05% Tween 20 (TBST) and incubated with 1 µL of anti-BLA poly-clonal primary antibody (Chemicon) in 3 mL of TBST for 10 min. For detection of MBP, 1 µL of anti-MBP poly-clonal primary antibody (Chemicon) was diluted in 3 mL of TBST and applied to the membrane for 10 min. After washing three times with 30 mL of TBST, the membrane was incubated with 0.5 µL of the secondary antibody (goat-antirabbit, Bio-Rad) in 3 mL TBST for 10 min followed by washing with 30 mL TBST 3 times. The blot was incubated with 1 mL of WesternC substrate and enhancer (Bio-Rad) for 3 min and imaged using a ChemiDoc XRS imaging system (Bio-Rad). Quantification of the intensity of bands corresponding to the amount of the proteins was performed using Quantity One software (Bio-Rad).

### Protein purification

For proteins containing an MBP domain, purification occurred via an amylose column. Plasmids pDIMC8-RHPh8, pDIMC8-RHPhc4, and pDIMC8-MBP317-347 were each transformed into *malE^−^ E. coli* strain PM9F′ for expression; pDIMC8-MBP was transformed into DH5α-E. To produce each protein, one liter ZYM-5052 media [21] containing 50 µg/ml Cm and 5 mM maltose was inoculated with 2% overnight LB culture and shaken at 37°C. The culture was induced with 300 µM IPTG when the OD_600 nm_ reached 0.6 and incubated at 25°C for 16 hours. Pelleted cells were resuspended in 20 mL 50 mM sodium phosphate and 250 mM NaCl and lysed by French press. The soluble fraction was recovered by twice spinning the lysate at 20,000 g for 30 min and filtering the supernatant through a 0.45 µm syringe filter. The protein was purified using an amylose affinity column (New England Biolabs). Protein was eluted with 10 mM maltose and was dialyzed for 48 hrs at 4°C against three liters of 100 mM NaCl, 50 mM sodium phosphate buffer in a 10,000 MWCO dialysis cassette (Pierce), changing the dialysis buffer after 24 hrs. Protein was concentrated in 20% glycerol, 100 mM NaCl, 50 mM sodium phosphate buffer using 10,000 MWCO spin columns (Amicon). Protein was stored in aliquots at −20°C.

For TEM-1 BLA, plasmid pDIMC8-BLA was transformed into DH5α-E. One liter ZYM-5052 media [21] containing 50 µg/ml Cm was inoculated with 5 mL overnight LB culture and shaken at 37°C. The culture was induced with 1 µM IPTG when the OD_600 nm_ reached 0.6 and incubated at 37°C for 16 hours. Pelleted cells were resuspended in 15 mL 50 mM sodium phosphate and 250 mM NaCl and lysed by French press. The soluble fraction was recovered and the protein was purified using an m-aminophenylboronic acid-agarose affinity column (Sigma). Protein was eluted with 0.5 M boric acid, 0.5 M NaCl, pH 7.0. Protein was concentrated and buffer-exchanged using 0.05 M Tris, 1.5 M NaCl and 10,000 MWCO spin columns (Amicon). Protein was then further purified using an Akta FPLC, with a HiPrep Q FF 16/10 anion exchange column. Protein was again concentrated and buffer exchanged with 50 mM phosphate buffer, 100 mM NaCl, 10% glycerol using 10,000 MWCO spin columns (Amicon). Protein was stored in aliquots at −20°C.

The purities of all proteins were estimated by coomassie blue staining of sodium dodecyl sulphate-polyacrylamide gel electrophoresis gels. The purities of Ph8, c4, MBP317-347 and MBP were greater than 95%. Protein concentrations were measured using a Nanodrop spectrophotometer at 280 nm.

### Enzyme kinetic characterization

Hydrolysis of ampicillin was measured at 37°C in the presence of 10 mM sodium phosphate buffer, pH 7.0. The concentrations of Ph8 and c4 were 15 nM and 2 nM, respectively. The concentration of substrate was varied from 100 µM to 400 µM. Enzyme stocks were incubated with or without maltose for 1 hr at 4°C. After a mixture of water, buffer and ampicillin had reached the desired temperature, 10 µL of enzyme was added to the cuvette and the absorbance at 240 nm was recorded using a Cary50 UV-VIS spectrophotometer. The initial rate of reaction was used to calculate the kinetic constants via Eadie-Hofstee plots. Although the calculated value of *K*_m_ for c4 indicates that the *K*_m_ for c4 could be more accurately determined using experiments with lower concentrations of ampicillin, the experiments as performed are sufficient for demonstrating the lack of effect of maltose on the kinetic parameters.

### Thermal unfolding

Denaturation as a function of temperature of MBP, BLA, MBP317-347, Ph8 and c4 was examined using circular dichroism (CD) spectroscopy. Protein samples were incubated with and without 5 mM maltose for 1 hr at 4°C prior to thermal denaturation. CD measurements at 222 nm were recorded using a Jasco J-710 CD Spectropolarimeter (Easton, MD). The denaturation measurements were carried out using a quartz cuvette (pathlength of 0.1 cm) in 30 mM sodium phosphate buffer (pH 7.0) at 0.5 mg/mL protein. CD absorbance was collected from 25–85°C at a temperature ramp rate of 1°C/min and a bandwidth of 1 nm. The background due to the buffer was measured and subtracted from the sample absorbance. For comparison of the results, the data were normalized and fitted to a two-state unfolding model [22].

## References

[1] Ostermeier M (2009). Designing switchable enzymes.. Curr Opin Struct Biol.

[2] Guntas G, Mansell TJ, Kim JR, Ostermeier M (2005). Directed evolution of protein switches and their application to the creation of ligand-binding proteins.. Proc Natl Acad Sci U S A.

[3] Guntas G, Mitchell SF, Ostermeier M (2004). A molecular switch created by in vitro recombination of nonhomologous genes.. Chem Biol.

[4] Guntas G, Ostermeier M (2004). Creation of an allosteric enzyme by domain insertion.. J Mol Biol.

[5] Sohka T, Heins RA, Phelan RM, Greisler JM, Townsend CA (2009). An externally-tunable bacterial band-pass filter.. Proc Natl Acad Sci USA.

[6] Goldsmith M, Kiss C, Bradbury AR, Tawfik DS (2007). Avoiding and controlling double transformation artifacts.. Protein Eng Des Sel.

[7] Raibaud O, Schwartz M (1980). Restriction map of the Escherichia coli malA region and identification of the malT product.. J Bacteriol.

[8] Sigal IS, DeGrado WF, Thomas BJ, Petteway SR (1984). Purification and properties of thiol beta-lactamase. A mutant of pBR322 beta-lactamase in which the active site serine has been replaced with cysteine.. J Biol Chem.

[9] Raquet X, Lamotte-Brasseur J, Fonze E, Goussard S, Courvalin P (1994). TEM beta-lactamase mutants hydrolysing third-generation cephalosporins. A kinetic and molecular modelling analysis.. J Mol Biol.

[10] Novokhatny V, Ingham K (1997). Thermodynamics of maltose binding protein unfolding.. Protein Sci.

[11] Vanhove M, Houba S, b1motte-Brasseur J, Frere JM (1995). Probing the determinants of protein stability: comparison of class A beta-lactamases.. Biochem J.

[12] Rosgen J, Pettitt BM, Bolen DW (2005). Protein folding, stability, and solvation structure in osmolyte solutions.. Biophys J.

[13] Rosgen J (2007). Molecular basis of osmolyte effects on protein and metabolites.. Methods Enzymol.

[14] Martineau P, Szmelcman S, Spurlino JC, Quiocho FA, Hofnung M (1990). Genetic approach to the role of tryptophan residues in the activities and fluorescence of a bacterial periplasmic maltose-binding protein.. J Mol Biol.

[15] Foit L, Morgan GJ, Kern MJ, Steimer LR, von Hacht AA (2009). Optimizing protein stability in vivo.. Mol Cell.

[16] Millet O, Hudson RP, Kay LE (2003). The energetic cost of domain reorientation in maltose-binding protein as studied by NMR and fluorescence spectroscopy.. Proc Natl Acad Sci U S A.

[17] Coombs GS, Bergstrom RC, Madison EL, Corey DR (1998). Directing sequence-specific proteolysis to new targets. The influence of loop size and target sequence on selective proteolysis by tissue-type plasminogen activator and urokinase-type plasminogen activator.. J Biol Chem.

[18] Hubbard SJ, Campbell SF, Thornton JM (1991). Molecular recognition. Conformational analysis of limited proteolytic sites and serine proteinase protein inhibitors.. J Mol Biol.

[19] Wright CM, Wright RC, Eshleman JR, Ostermeier M (2011). A protein therapeutic modality founded on molecular regulation.. Proc Natl Acad Sci U S A.

[20] Betton J-M, Hofnung M (1994). *In vivo* assembly of active maltose binding protein from independently exported protein fragments.. EMBO J.

[21] Studier FW (2005). Protein production by auto-induction in high density shaking cultures.. Protein Expr Purif.

[22] Pace CN (1986). Determination and analysis of urea and guanidine hydrochloride denaturation curves.. Methods Enzymol.

